# KPC-3-, GES-5-, and VIM-1-Producing *Enterobacterales* Isolated from Urban Ponds

**DOI:** 10.3390/ijerph19105848

**Published:** 2022-05-11

**Authors:** Pedro Teixeira, Nuno Pinto, Isabel Henriques, Marta Tacão

**Affiliations:** 1 CESAM (Centre for Marine and Environmental Studies), University of Aveiro, 3810-193 Aveiro, Portugal; pedrofteixeira@ua.pt (P.T.); martat@ua.pt (M.T.); 2 Biology Department, University of Aveiro, 3810-193 Aveiro, Portugal; vod4gnunop@gmail.com; 3 Centre for Functional Ecology, Department of Life Sciences, University of Coimbra, 3004-531 Coimbra, Portugal

**Keywords:** antibiotic resistance, carbapenemases, *Enterobacterales*, urban aquatic environments

## Abstract

Carbapenems are antibiotics of pivotal importance in human medicine, the efficacy of which is threatened by the increasing prevalence of carbapenem-resistant *Enterobacterales* (CRE). Urban ponds may be reservoirs of CRE, although this hypothesis has been poorly explored. We assessed the proportion of CRE in urban ponds over a one-year period and retrieved 23 isolates. These were submitted to BOX-PCR, PFGE, 16S rDNA sequencing, antibiotic susceptibility tests, detection of carbapenemase-encoding genes, and conjugation assays. Isolates were affiliated with *Klebsiella* (*n* = 1), *Raoultella* (*n* = 11), *Citrobacter* (*n* = 8), and *Enterobacter* (*n* = 3). Carbapenemase-encoding genes were detected in 21 isolates: *bla*_KPC_ (*n* = 20), *bla*_GES-5_ (*n* = 6), and *bla*_VIM_ (*n* = 1), with 7 isolates carrying two carbapenemase genes. Clonal isolates were collected from different ponds and in different campaigns. *Citrobacter* F6, *Raoultella* N9, and *Enterobacter* N10 were predicted as pathogens from whole-genome sequence analysis, which also revealed the presence of several resistance genes and mobile genetic elements. We found that *bla*_KPC-3_ was located on Tn*4401*b (*Citrobacter* F6 and *Enterobacter* N10) or Tn*4401*d (*Raoultella* N9). The former was part of an IncFIA-FII pBK30683-like plasmid. In addition, *bla*_GES-5_ was in a class 3 integron, either chromosomal (*Raoultella* N9) or plasmidic (*Enterobacter* N10). Our findings confirmed the role of urban ponds as reservoirs and dispersal sites for CRE.

## 1. Introduction

The spread of antimicrobial resistance and the upsurge in multidrug-resistant bacteria over the last decades led to an unprecedented impact on public health systems all around the globe, causing an ever-rising death toll accompanied by a significant economic impact [1,2,3]. Moreover, therapeutic options used to treat infectious diseases are becoming increasingly scarce, and even last-resort antibiotics are losing their efficacy [4].

Carbapenems, which are among the most active and potent agents against multidrug-resistant Gram-negative pathogens, are becoming threatened by the global dissemination of carbapenem-resistant bacteria, in which carbapenem-resistant *Enterobacterales* (CRE) play an important role [5]. As a result, the World Health Organization (WHO) has included CRE in the list of critical targets for research and development of new antibiotics [6]. The two main mechanisms behind carbapenem resistance in CRE consist of the expression of cephalosporinases combined with mutation-derived permeability defects and, even more important, in carbapenemase production, enzymes that are able to efficiently hydrolyze carbapenem antibiotics [7]. KPC appears to dominate over other carbapenemases in Portugal, being associated with significant outbreaks of both KPC-2 and KPC-3 variants [8,9,10]. Carbapenemase-producing bacteria have also been isolated from several different Portuguese environmental settings, namely river water (IMP-, VIM-, KPC-, GES-, and NDM-producing strains) [11,12,13], wastewater (KPC-producing) [14], urban streams (GES-producing) [15], and wildlife (OXA-48-, GES- and KPC-producing) [16]. Carbapenemase-coding genes are usually located in association with mobile genetic elements (MGEs) that enable their spread through horizontal transfer [17]. For instance, the *bla*_KPC_ gene has already been described in many different conjugative plasmids, and the transfer between these structures has been potentiated by the Tn*3*-based composite transposon Tn*4401* [11,12,14,16,18]. In turn, *bla*_GES_ genes are often associated with integrons within plasmids that carry other antibiotic-resistance genes (ARGs) [11,19,20].

Currently, the role of aquatic environments in the accumulation and dissemination of antibiotic-resistant bacteria and ARGs, including CRE and carbapenemase-encoding genes, is well recognized [21,22]. In parallel, the surge in the application of the One Health approach has highlighted the link between anthropogenic contamination and the accumulation of antibiotic resistance in environmental habitats [23,24].

Lately, the importance of rivers within the environmental resistome framework has been evidenced, given their role in transporting resistant bacteria and ARGs and the fact that intense human activity in these ecosystems enables exposure to these bacteria and genes [11,21,25]. However, other aquatic systems such as urban lakes and ponds, which have been much less studied, are also extensively impacted by human activities and may constitute hotspots of antibiotic resistance [26]. These systems consist of natural or artificial inland bodies of surface water, surrounded by an urban environment and therefore often used for recreational purposes [27,28]. Unlike in rivers, resistant pathogens and ARGs undergo longer retention times in urban lakes and ponds [29], and the high bacterial density can promote the horizontal transfer of ARGs, enhancing the emergence of multidrug-resistant strains [30,31]. However, despite the potential role of urban lakes and ponds to accumulate and spread antibiotic-resistant bacteria and ARGs, only recently have these aquatic systems begun to be explored. A few studies have revealed a high abundance and diversity of ARGs and MGEs, as well as the occurrence of antibiotic-resistant human pathogens. CRE and carbapenemase coding genes have been sporadically detected [29,32,33,34,35], but the available information is clearly insufficient to understand the potential risks associated with these environments. In addition, although the seasonal dynamics of antibiotic resistance has been accessed in these systems [36,37], to best of our knowledge, there is no information on the ability of CRE to persist in urban ponds over time.

The aim of this study was to: (i) assess the occurrence of CRE in water samples obtained from urban aquatic systems located in the urban area of Aveiro (Portugal); (ii) understand which mechanisms of carbapenem resistance are present in environmental CRE; and (iii) characterize CRE isolates in terms of antibiotic resistance, genetic platforms associated with carbapenemase genes, plasmid content and other MGEs, and virulence traits.

## 2. Materials and Methods

### 2.1. Sample Collection and Isolation of CRE

**Figure 1:**
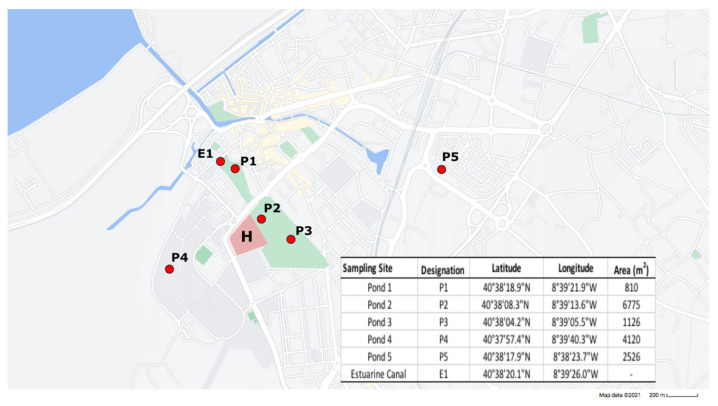
Map depicting the location, designation and coordinates of the sampled ponds (P1–P5) and estuarine canal (E1). Green areas indicate the location of urban parks, while blue areas represent the Ria de Aveiro urban canals. The location of the municipal hospital is also denoted (H).

Between February 2019 and February 2020, surface water samples were collected from five ponds (P1–P5) located within or in close proximity to urban parks in Aveiro, a city that comprises about 55,000 inhabitants (Figure 1). 

Samples from an estuarine canal (E1), located directly downstream from Pond 1, were also included to investigate a possible connection between these systems. Due to logistic constraints, in both February and March 2019, samples were only collected from ponds P1 and P2, while in September 2019, no samples were collected from the estuarine canal E1. Samples were collected in sterile bottles and stored at 4 °C until analysis. Water samples were filtered in triplicate through mixed cellulose ester membranes (GN-6 Metricel^®^) with a 0.45 μm pore size. The filter membranes were then transferred to m-Fecal Coliform agar (m-FC) (VWR) plates supplemented with imipenem (4 μg/mL) and incubated at 37 °C for 18 h. To estimate the proportion of imipenem-resistant bacteria, m-FC without antibiotic was used. Coliform colonies were counted, and imipenem-resistant colonies were purified and stored in 20% glycerol at −80 °C.

### 2.2. Molecular Typing and Identification

Whole-cell suspensions were used as the DNA template for each isolate in all PCR analysis. Briefly, one bacterial colony of each isolate was resuspended in 20 µL of sterile distilled water, and 1 µL of this suspension was used as template in each PCR reaction. BOX-PCR (primers and conditions as described in Tacão et al., 2012 [38]) was used as a first discriminatory molecular-typing method for imipenem-resistant isolates. Isolates recovered from each sampling site exhibiting different BOX-profiles were selected for amplification and analysis of the 16S rRNA gene using the primers listed in Table S1. Additionally, considering the lack of accuracy of most phenotypic tests in the identification of *Raoultella* species, all isolates affiliated with the genus *Raoultella* were further identified using matrix-laser desorption ionization mass spectrometry (Bruker MALDI Biotyper IVD, Portugal) in order to obtain a reliable species identification [39].

CRE isolates with different BOX profiles or collected in different sampling campaigns and from different ponds were further analyzed by PFGE of XbaI-digested DNA, using the CHEF-DR II System (Bio-Rad Laboratories, Hercules, CA, USA) as previously described [40]. PFGE patterns were analyzed using GelCompar II software (v 6.1) with the criteria established by Tenover and colleagues [41].

### 2.3. Antibiotic Susceptibility Testing and Screening of ARGs

To estimate the resistance levels of the CRE isolates, antimicrobial resistance was tested by the disk-diffusion method, in agreement with the European Committee on Antimicrobial Susceptibility Testing (EUCAST) [42]. The following panel of 16 antibiotic was used: amikacin (AK), aztreonam (ATM), cefepime (FEP), cefotaxime (CTX), ceftazidime (CAZ), ciprofloxacin (CIP), chloramphenicol (C), ertapenem (ETP), gentamicin (CN), imipenem (IPM), meropenem (MEM), piperacillin (PRL), piperacillin/tazobactam (TZP), sulfamethoxazole/trimethoprim (SXT), tetracycline (TE), and tigecycline (TGC) (Oxoid). Quality control was performed using *Escherichia coli* ATCC 25922. EUCAST clinical breakpoints (version 10.0, 2020) were used to classify the CRE isolates as susceptible, intermediate, or resistant. Isolates were screened for the presence of carbapenemase-encoding genes (*bla_IMP_*, *bla_VIM_*, *bla_KPC_*, *bla_GES_*, *bla_NDM_,* and *bla_OXA-48_*) and integron coding genes of classes 1, 2, and 3 using primers previously described (Table S1)**.** Isolates were also tested using PCR for the presence of the colistin-resistance gene *mcr-1* and the ESBL-coding gene *bla*_CTX-M_ (Table S1), given the clinical relevance of these genes [43,44] and their previous detection in CRE [45,46].

### 2.4. Mating Assays and Plasmid Analysis

Mating assays were performed for CRE harboring carbapenemase genes that exhibited unique PFGE patterns, as previously described [47]. In short, donor strains and the sodium-azide-resistant strain *E. coli* J53 (recipient strain) were grown overnight in Luria–Bertani broth (LB) at 37 °C and 180 rpm. Donor and recipient strains were mixed at a 1:1 ratio on solid media using plate count agar (PCA) and left overnight at 37 °C. Putative transconjugants were selected on PCA plates supplemented with imipenem (4 μg/mL) and sodium azide (100 μg/mL). Transconjugants identity was confirmed with BOX-PCR typing and PCR confirmation of the acquired carbapenemase gene. Minimum inhibitory concentration (MIC) gradient strips (Liofilchem, Italy) were used to evaluate the susceptibility of donors, transconjugants, and the recipient strain to imipenem, meropenem, ertapenem, ceftazidime, and cefotaxime, following the EUCAST MIC clinical breakpoints [42].

Plasmid DNA was extracted using the NZYMiniprep kit (NZYTech, Portugal) following the manufacturer’s protocol and analyzed by electrophoresis. Plasmid replicon typing (PBRT) was performed to detect replicons belonging to 18 incompatibility groups, as previously described [48]. We also evaluated the presence of pBK30661- and pBK30683-like plasmids, as described before [49].

### 2.5. Whole-Genome Sequence Analysis

Three CRE isolates were selected for whole-genome sequence (WGS) based on molecular typing and both antibiotic-resistance phenotype and genotype. Genomic DNA was extracted from each isolate using the Wizard^®^ Genomic DNA Purification Kit (Promega, Madison, WI, USA) according to the manufacturer’s protocol. Paired-end libraries were created using the Illumina HiSeq 2500 platform. The raw reads quality was verified using FastQC software and submitted to the trimming process in order to exclude those that contained a phred quality score below 20 using Trimmomatic v.0.36 [50]. The genomes were assembled using the SPAdes version 3.14.0 program [51]. Draft genomes were annotated using Rapid Annotation using Subsystem Technology (RAST) [52]. The rRNA were predicted using the RNAmmer 1.2 Server [53], and tRNA were predicted using tRNAscan-SE 2.0 [54]. In order to confirm species identification, ANIb and ANIm were calculated by using the JSpeciesWS tool [55], and dDDH was calculated against representative genomes by using the Genome-to-Genome Distance Calculator v2.1 [56]. We also considered the G + C% divergence in the overall genome-relatedness analysis [57]. Sequence types were attributed through a multilocus sequence typing analysis using MLST v2.0 [58] and PubMLST v1 [59]. ARGs were screened using CARD [60], and genomic islands were predicted using the Genomic Island Prediction Software (GIPSy) [61]. In silico screening of plasmid replicons was performed using PlasmidFinder v2.1 [62]. The CRISPR Finder tool [63] and the PHASTER tool [64] were used to assess the presence of clustered regularly interspaced short palindromic repeats (CRISPRs) and prophage sequences. Putative virulence factors were inspected using the Virulence Factors of Pathogenic Bacteria (VFDB) database [65]. The pathogenicities of all three CRE isolates were also accessed using PathogenFinder 1.1 [66]. 

## 3. Results

### 3.1. Prevalence and Diversity of CRE in Urban Ponds

Colony counts on mFC varied between 0.5 and 3.70 log_10_CFU/mL (Figure S1). Globally, P1 exhibited the highest average mean mFC counts/mL (3.02 log_10_), while P4 exhibited the lowest (1.05 log_10_). The February 2019 campaign was associated with overall higher mFC counts/mL, followed by December and March 2019.

Imipenem-resistant bacteria were detected in ponds P1, P2, and P3, and also in the estuarine canal E1. P1 and E1 were the only aquatic systems in which imipenem-resistant colonies were detected in all sampling campaigns. On the other hand, in pond P2, imipenem-resistant colonies were detected only in February and March 2019, and in pond P3 only in November 2019. Overall, the percentage of imipenem-resistant CFUs was low (0.021% on average), with the highest percentage (0.16%) occurring in site P1 in September 2019 (Table S2). A collection of 23 CREs was obtained in this study, isolated from sites P1 (*n* = 19), P3 (*n* = 2), and E1 (*n* = 2). Isolates were affiliated with the genera *Citrobacter* (*n* = 8), *Raoultella* (*n* = 11), *Enterobacter* (*n* = 3), and *Klebsiella* (*n* = 1) (Table 1). The MALDI-TOF analysis of the *Raoultella* isolates showed that they all were affiliated with *Raoultella ornithinolytica*. The 16S rRNA gene sequence analysis of the imipenem-resistant bacteria found in Pond 2 demonstrated their affiliation with the *Shewanella* genus, which harbor intrinsic carbapenem-resistance mechanisms [67], and were consequently excluded from the CRE collection. 

### 3.2. Antibiotic Susceptibility, ARGs and MGEs Content

The majority of the CRE collection was classified as multidrug resistant (approximately 87%), with the only exceptions being *Enterobacter* isolates (*n* = 3). The isolates were resistant to all β-lactams (Table 1), with the exception of the *Klebsiella* isolate, which was susceptible to cefepime and aztreonam. On the other hand, the CRE isolates were susceptible to tigecycline, with the exception of isolates *Citrobacter* F1 and F4. Additionally, the *Enterobacter* isolates were susceptible to all non-β-lactam antibiotics tested, excluding E*nterobacter* N10, which was resistant to ciprofloxacin. Within the *Raoultella* genus, all isolates were susceptible to tetracycline, tigecycline, and chloramphenicol.

With the exceptions of *Enterobacter* N11 and *Enterobacter* N15, CRE isolates harbored at least one of the inspected carbapenemase-encoding genes (Table 1); *bla*_KPC_ was the most frequently identified (20 out of 23), being detected in all *Raoultella* and *Citrobacter* isolates, and also in *Enterobacter* N10. Furthermore, the *Enterobacter* N10 and *Raoultella* isolates (*n* = 5) collected in November 2019 coharbored both *bla*_KPC_ and *bla*_GES._ The sequencing analysis revealed the presence of the *bla*_GES-5_ variant, which has been proven to exhibit carbapenemase activity [68]. The *bla*_VIM_ gene was only detected in *Citrobacter* N5, which also harbored *bla*_KPC_. The single *Klebsiella* isolate in the collection harbored *bla*_GES-5_. Both *bla*_CTX-M_ and *mcr-1* genes were not detected.

The CRE collection was also inspected for the presence of integrons. The gene *IntI1* was detected in all CRE isolates, except for *Enterobacter* N11, *Enterobacter* N15, and *Klebsiella* N14. *IntI3* was identified in *Klebsiella* N14. Moreover, with the exception of *Raoultella* N1, all isolates that were collected in November 2019 in which both *bla*_KPC_ and *bla*_GES-5_ were detected also coharbored both integrase-encoding genes *IntI1* and *IntI3*. Class 2 integrons were not detected (Table 1).

The genetic context of the *bla*_VIM_ gene identified in *Citrobacter* N5 was determined by PCR and sequence analysis, revealing the presence of this gene in a class 1 integron containing other ARGs encoding resistance to aminoglycosides (*aacA4*; *aadA1*), chloramphenicol (*catB2*), and sulfonamides (*sul1*). Further PCR analysis also revealed the presence of the *bla*_GES-5_ gene cassette as part of a class 3 integron in all isolates in which this gene was detected.

### 3.3. CRE Clonal Relatedness

Clonal relationships among the CRE isolates were first assessed using BOX-PCR (Table 1). Among the *Citrobacter* genus, the same BOX profile was observed within a group comprising isolates collected in February 2019 and N6 (collected in November 2019). In regard to the *Enterobacter* genus, both isolates N11 and N15, collected from Pond 3, shared the same BOX profile; while isolate N10, collected from Pond 1, exhibited a singular BOX profile. Within the *Raoultella* genus, two clusters exhibiting two distinctive BOX profiles were observed, one composed of all *Raoultella* isolates collected from Pond 1 in September and October 2019, and the other composed of all *Raoultella* isolates collected in November 2019 from sampling sites P1 and E1. Although we adopted the criteria of different BOX profiles and/or sampling campaigns/sites in the selection of CRE for PFGE analysis, isolates obtained from sampling site E1 (N12 and N13) were both selected due to the PCR detection of pBK30683-like plasmid exclusively on *Raoultella* N12.

The PFGE analysis further divided *Raoultella* isolates into two different clusters (Figure S2). One of the clusters comprised isolates collected in November 2019, with isolate N12 exhibiting a slightly different PFGE profile. The other cluster was composed of *Raoultella* isolates collected in October 2019 and isolate S1 from the September campaign. Regarding the *Citrobacter* genus, PFGE analysis resulted in two singletons represented by N5 and F5 (Figure S2), and one cluster comprising two separate clades with isolates collected in February and November. Together, the results obtained with BOX and PFGE confirmed the presence of clonal isolates in different ponds (*Raoultella* N1, N8, and N9 in pond P1 and *Raoultella* N12 in pond E1) and collected in different campaigns (*Raoultella* S1 and O1 in September and October 2019, respectively; *Raoultella* N1 in November 2019 and *Raoultella* N8, N9, and N12 in November 2020; and finally *Citrobacter* F1 and N6 in February 2019 and November 2020, respectively).

### 3.4. Plasmid Analysis and Transfer of Carbapenem Resistance

The presence of plasmids was evaluated in all CRE isolates (Figure S3), and 10 distinct plasmid profiles were identified. Within the *Citrobacter* genus, four distinctive plasmid profiles were detected. Regarding the *Raoultella* genus, all isolates collected in November exhibited the same plasmid profile. *Raoultella* O5 exhibited a singular plasmid profile, while *Raoultella* O1 and all isolates collected in September shared the same plasmid profile. Among the *Enterobacter* isolates, two distinct plasmid profiles were identified. The single *Klebsiella* isolate (N14) also showed a unique plasmid profile.

Two known plasmid replicons were detected by PBRT, namely IncL/M and IncN (Table 1). IncL/M was detected in *Enterobacter* N10 and both *Citrobacter* F4 and F6, while IncN was detected in *Citrobacter* isolates F3, F6, N5, and N6. Additionally, both replicons were detected in *Citrobacter* F6. The IncFIA-FII pBK30683-like plasmid structure was present in *Raoultella* isolates N1, N9, and N12 (Table 1).

The mating assays resulted in two transconjugants from *Citrobacter* isolates F1 and N6. Transconjugants carrying the *bla*_KPC_ gene exhibited significant MIC increases for carbapenems (from to 5 to 62 times) and cephalosporins (from 187 to 680 times) (Table 2). Transfer frequencies (transconjugants per recipient cell) of *bla*_KPC_-harboring plasmids to *E. coli* J53 were 1.56 × 10^−4^ for *Citrobacter* F1 and 5.18 × 10^−4^ for *Citrobacter* N6.

### 3.5. WGS Analysis

Three CRE isolates representing the three most represented genera were selected for WGS and further analysis: *Citrobacter* F6, *R. ornithinolytica* N9, and *Enterobacter* N10. The general genomic features of all three genomes are described in detail in Table S3.

Whole-genome sequence-based methods (ANIb, ANIm, dDDH, and G + C difference) were congruent in the identification of *Citrobacter* F6 as *Citrobacter freundii*, *Raoultella* N9 as *Raoultella ornithinolytica,* and Enterobacter N10 as *Enterobacter kobei* (Table S3). Furthermore, all three strains were predicted as human pathogens by PathogenFinder.

#### 3.5.1. *C. freundii* F6

The *C. freundii* F6 draft genome was 5,117,230 bp in size and organized in 230 contigs, with 51.7% in terms of G + C content, an N50 of 88,228, and 4997 predicted coding sequences (Table S3). *C. freundii* F6 belonged to the sequence type ST270 (Table S3), and based on WGS analysis, a total of 142 virulence factors were predicted; they were distributed in a total of 16 virulence categories, with most allocated to secretion systems (*n* = 33), antiphagocytosis (*n* = 22), and adherence (*n* = 17) functions (Figure S5).

In addition to *bla*_KPC-3_, the ARGs identified on *C. freundii* F6 consisted of the Amp-C β-lactamase-coding gene *bla*_CMY-152_, *dfrA14* (trimethoprim resistance), *tetA* (tetracycline resistance), and *qnrS1* (quinolone resistance), the latter being commonly present in plasmid structures [69] (Table 3).

In fact, a BLAST search revealed that the contig in which *qnrS1* was located (3259 bp in size) exhibited 100% similarity in terms of nucleotide identity with part of a 249.6 Kb plasmid previously described in a *Salmonella enterica* isolate (CP039857). Additional ARGs, namely *bla*_OXA-1_ (encoding β-lactam resistance), *catB3* (chloramphenicol resistance), *aacA4-cr* (aminoglycoside resistance), *mphA* (encoding macrolide resistance), and *sul1* (encoding sulfonamide resistance) (Table 3) were found within a class 1 integron (In1387) previously described in the IncN3 broad-host-range plasmid pTRE-132 [70], exhibiting the following gene cassette array: 5′CS *IntI1*|*aacA4-cr*|*bla*_OXA-1_|*catB3*|*qacΔ1*|*sul1*. Botts and colleagues captured the pTRE-132 plasmid from wetland bacteria into an *E. coli* recipient strain that was able to further transfer it through conjugation, resulting in resistance to ampicillin and ticarcillin, as well as decreased susceptibility to other classes of antibiotics (fluoroquinolones, chloramphenicol, aminoglycosides, and sulfonamides) [70]. This plasmid also harbors other ARGs, namely *qnrB* and an additional copy of *sul1* [70].

In terms of MGEs, six replicons were detected in *C. freundii* F6, namely pKPC-CAV1321, IncFIA (HI1), IncFII (K), IncM1, and IncN (Table 3). Further genomic analysis led to the identification of 14 putative pathogenicity islands, 6 putative resistance islands, 2 CRISPR sequences, and 5 intact prophage regions (Figure S4). The sequence analysis of these genomic islands identified coding sequences that were part of a mercury-resistance operon, a type IV secretion system, and multidrug-efflux transporters responsible for the transport of metals.

**Figure 2:**
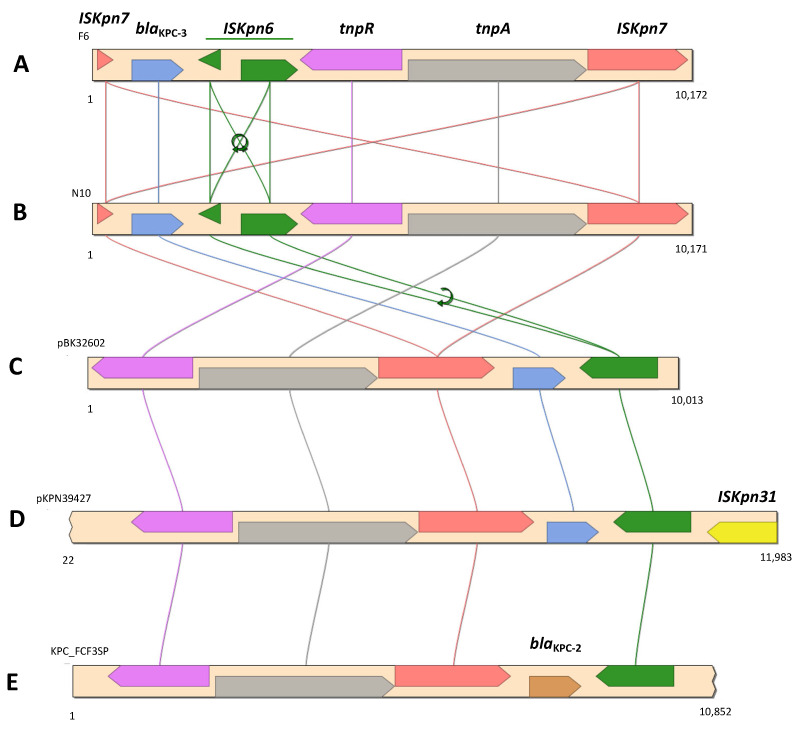
Genetic context of bla_KPC-3_ in *C. freundii* F6 (**A**), *E. kobei* N10 (**B**), *E. coli* BK32602 (**C**) (KU295134), *K. pneumoniae* strain 39427 (**D**) (CP054266), and *K. pneumoniae* FCF3SP (**E**) (CP004367).

Next, *bla*_KPC-3_ was identified by GIPSy as being part of resistance island RI5 (Figure S4A). In addition to *bla*_KPC-3_, RI5 also harbored a gene coding for a multidrug-efflux transporter. The carbapenemase-encoding gene was flanked by two insertion sequences, *ISKpn6* and *ISKpn7*, which in turn were part of a Tn*4401*b transposon (Figure 2).

The Tn*4401*b isoform was confirmed, as no deletions were observed in the 386 bp region [71]. However, both *ISKpn6* and *ISKpn7* were fragmented (Figure 2). The *bla*_KPC-3_-harboring contig (10,172 bp) revealed the same nucleotide sequence (100% identity; query coverage of 98%) with the corresponding region of an IncN plasmid described in a clinical *E. coli* strain (KU295134), and 99.7% of nucleotide sequence similarity (query coverage of 97%) with one other IncN plasmid described in several *K. pneumoniae* clinical strains [72], and also with a *IncFIB/FII* plasmid identified in a *K. pneumoniae* clinical strain (CP054266) [73].

#### 3.5.2. *R. ornithinolytica* N9

The *R. ornithinolytica* N9 draft genome was 6,252,962 bp in size and organized in 420 contigs, with 55.2% in terms of G + C content, an N50 of 109,201, and 6014 predicted coding sequences (Table S3). A total of 130 virulence factors were predicted for *R. ornithinolytica* N9 across 12 virulence categories, with adherence (*n* = 44), iron uptake (*n* = 34), and secretion systems (*n* = 20) being the most represented (Figure S5).

The genomic analysis of *R. ornithinolytica* N9 confirmed the presence of a cointegrated IncFIA/FII plasmid, designated pN9KPC, exhibiting 99.93% similarity in terms of the nucleotide sequence with pBK30683 (KF954760), previously identified with the PCR-based protocol (Table 3) [49]. In addition to *bla*_KPC-3_, other resistance determinants previously reported in pBK30683-like plasmids were detected, including *bla*_TEM-1_, *bla*_OXA-9_ (encoding β-lactam resistance), *aacA*4, *aadA1*, *strB*, *strA* (aminoglycoside resistance), *sul2* (sulfonamide resistance), and *dfrA14* (trimethoprim resistance), which were also detected in pN9KPC (Table 3). The *dfrA14* gene was part of the putative resistance island RI3 located in the same plasmid. Within this plasmid, the *bla*_KPC-3_ gene was located on a Tn*4401*d transposon, flanked by the insertion sequences *ISKpn6* and *ISKpn7*, as previously described [49].

The analysis of the *bla*_GES-5_ gene context revealed its location in a class 3 integron, followed by part of an *aacA4* gene cassette. However, the contig ended at this cassette, and it was not possible to clarify the full composition of this integron. A gene encoding an ORN β-lactamase, *bla*_ORN-1_, intrinsic in *R. ornithinolytica* chromosome [74], was also detected on the same contig of *bla*_GES-5_.

Other resistance determinants were found in the N9 genome, including *bla*_OXA-10_ (β-lactam resistance), *fosA* (fosfomycin resistance), and the AmpC β-lactamase-encoding gene *bla*_MOX-3_, which was located in the same contig as a class 1 integrase and the ARGs *msrE* and *mphE* (macrolide resistance) (Table 3).

In addition to pN9KPC, three other additional replicons were detected in *R. ornithinolytica* N9, namely Col(MGD2), Col(pHAD28), and Col440I (Table 3). A total of 13 putative pathogenicity islands, 3 putative resistance islands, 7 CRISPR sequences, and 6 intact prophage regions were identified (Figure S4).

#### 3.5.3*. E. kobei* N10

The predicted *E. kobei* N10 draft genome was 5,397,864 bp in size and organized in 431 contigs, with 54.4% in terms of G+C content, an N50 of 65,421, and 5128 predicted coding sequences (Table S3). *E. kobei* N10 was assigned to the new sequence type ST1378, as a result of the new alleles detected in *fusA*, *leuS,* and *rpiB* genes (Table S3). A total of 152 virulence factors were predicted for *E. kobei N10*, distributed over 15 virulence categories and with secretion systems (*n* = 45), iron uptake (*n* = 26), and antiphagocytosis (*n* = 24) being the most represented (Figure S5).

Apart from *bla*_KPC-3_ and *bla*_GES-5_, other ARGs were also detected in *E. kobeii* N10, specifically the AmpC β-lactamase-encoding gene *bla*_ACT-9_, *aacA4*, *mphA*, *sul1,* and *fosA* (fosfomycin resistance) (Table 3).

Overall, six replicons were detected by PlasmidFinder, namely pKPC-CAV1321, *CoI440I*, *IncFIB*, IncM1, *IncN,* and *IncX5* (Table 3). A total of 17 putative resistance islands and 21 putative pathogenicity islands were detected in the GIPSy analysis, as well as 2 CRISPR sequences and 3 intact prophage regions (Figure S4).

**Figure 3:**
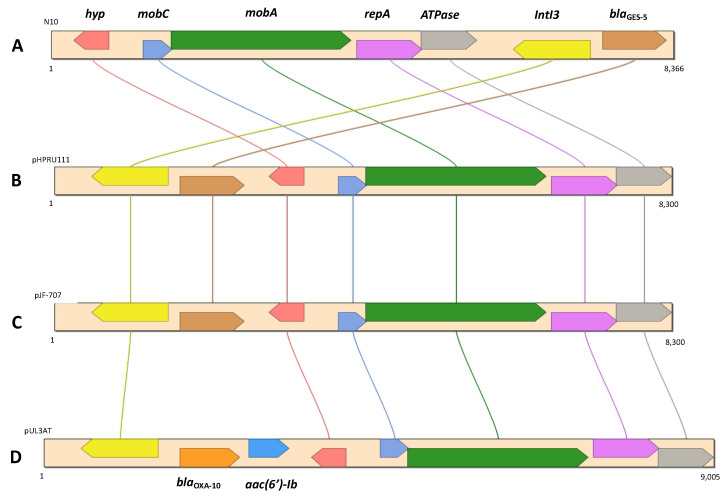
Genetic context of bla_GES-5_ in *E. kobei* N10 (**A**), *E. cloacae* Ecl-W (**B**) (MN180807), *K. oxytoca* strain H143640707 (**C**) (KX946994), and *E. cloacae* LIM73 (**D**) (HE616889).

Next, *bla*_GES-5_ was identified as part of a putative resistance island (RI17), as well as the *fosA* coding for fosfomycin resistance (RI7). We also found that *bla*_GES-5_ was present in a class 3 integron, which in turn was located in a contig (8366 bp) approximately 99.8% identical in terms of nucleotide identity to a corresponding region in plasmids pHPRU11 (MN180807), described in the clinical strain *E. cloacae* Ecl-w [75]; pJF-707 (KX946994), found in the clinical strain *K. oxytoca* H143640707; and pUL3AT (HE616889), found in *E. cloacae* LIM73 isolated from hospital effluent [76] (Figure 3). 

On the other hand, the *bla*_KPC-3_ gene was identified in a Tn*4401*b transposon, which in turn was located in a contig (10.17 Kb) that was 100% identical in terms of nucleotide sequence to the same region of the *C. freundii* F6 plasmid described above (Figure 3). These two isolates also had in common the presence of the ARGs *aacA4*, *mphA,* and *sul1*.

## 4. Discussion

Although we are witnessing an increase in the global levels of carbapenem resistance, the number of studies investigating the occurrence of CRE in urban lakes and ponds is still limited. In Portugal, reports on the occurrence of CRE in aquatic environments are still very scarce, and are limited to rivers [11,12,13,76] and wastewater effluents [14,19,77].

Urban lakes and ponds can serve as significant reservoirs of antibiotic-resistant bacteria and ARGs, and contribute to their spread due to the likely exposure of humans and animals; for example, during recreational activities [26,78]. On the other hand, in some cases these systems are physically connected to other aquatic systems in which the spread of antibiotic resistance can occur by other routes, such as estuarine channels. In this study, we reported the presence and characterization of CRE collected from five urban ponds and an estuarine canal located in the Aveiro city center.

Overall, the colony counts on mFC media (Figure S1) never exceeded the value corresponding to a fair classification of superficial waters (third-best classification possible out of five categories that varied from excellent to very poor) in terms of total coliforms, according to the criteria used by the National System of Information on Hydric Resources (SNIRH) to classify superficial water (https://snirh.apambiente.pt, accessed on 13 December 2021). The low percentage of imipenem-resistant *Enterobacterales* (between 0 and 1%) was not surprising, as carbapenems are considered last-resort drugs that are used exclusively in clinical settings in Portugal [76]. Pond 1 stood out from the other sampling sites as the only site where imipenem-resistant *Enterobacterales* were detected in every sampling campaign (Table S2). In fact, roughly 84% of the CRE collection was obtained from Pond 1 (Table 1). This pond is located in Aveiro Municipal Park, where several contamination sources could be responsible for the higher CRE prevalence. CRE present in the fecal droppings from companion animals have been previously described [79,80], although with low prevalence. On the other hand, runoffs from surrounding areas or leaks in wastewater pipelines also represent plausible contamination sources [14,81], including from hospital wastewater [82], considering that the hospital is located approximately 600 m from Pond 1, and the hospital’s wastewater pipeline system is also located in close proximity. However, further research is needed to clarify the contamination source. Molecular typing revealed that *Citrobacter* and *Raoultella* isolates exhibiting the same BOX and PFGE profiles could be detected in Pond 1 over time spans of 10 months and 1 month, respectively. The presence over a long period of the same strains may indicate the existence of a persistent contamination source that is responsible for periodic discharges. At the same time, CRE occurring in these ponds are not submitted to any streams or currents, in contrast to rivers and larger aquatic systems. Therefore, without a significant water flow regime, CRE are not exposed to the resulting dilution effect, and tend to accumulate [78]. The occurrence of *Raoultella* isolates with the same PFGE pulsotype in Pond 1 and in the adjacent E1 canal indicated that P1 may be contributing to the dissemination of CRE to the canal and ultimately to Ria de Aveiro, in which the occurrence of *Enterobacterales* harboring integrons and β-lactamase genes has been previously reported [83]. This hypothesis points to an even more worrying scenario, since Ria de Aveiro is used as a food source, therefore providing an additional route through which humans can be exposed to CRE.

The β-lactam resistance observed in the CRE collection was mainly due to the presence of the carbapenemase genes *bla*_KPC-3_ and *bla*_GES-5_. The high frequency of *bla*_KPC_ harboring CRE reflected the epidemiology observed in Portuguese hospitals, since it was the most frequent carbapenemase gene among CRE clinical isolates [9,84,85]. Furthermore, KPC was also the most common carbapenemase reported from environmental CRE in Portugal [11,12,14]. In contrast, Araújo and colleagues established a CRE collection obtained from raw wastewater in which *bla*_GES-5_ was detected in all isolates, including *Citrobacter* and *Enterobacter* isolates [19]. In addition, the occurrence of a GES-5-producing *K. pneumoniae* isolate recovered from an urban water stream has been described in Portugal [15]. As hypothesized by Araújo and colleagues [19], GES-5-producing CRE may originate from nonhuman sources.

*Citrobacter* species are known for their capacity to colonize humans, animals, plants, and the environment, and also are often associated with high-risk transferable ARGs such as carbapenemases [86]. The occurrence of environmental *bla*_KPC_-harboring *Citrobacter* has been previously described in rivers [25,87] and hospital effluents [88,89,90]. On the other hand, the copresence of *bla*_KPC_ and *bla*_VIM_ in *Citrobacter* has only been described once in a clinical *C. freundii* isolate in Spain [91]. In both the Spanish *C. freundii* clinical isolate and *Citrobacter* N5, *bla*_VIM-1_ was inserted in a class 1 integron containing five other ARGs: *aacA4*-*dfrB1*-*aadA1*-*catB3*-*sul1*, thus contributing to its MDR phenotype. *C. freundii* F6 belonged to sequence type ST270, which was first assigned to an isolate obtained from a diarrheal patient in China [92], showing that this ST is not confined to clinical settings, as it is disseminated across different continents.

In *C. freundii* F6, *R. ornithinolytica* N9, and *E. kobei* N10, the *bla*_KPC-3_ gene was present as part of a Tn*3*-based mobile transposon Tn*4401* exhibiting two different isoforms: Tn*4401*b in *C. freundii* F6 and *E. kobei* N10; and Tn*4401*d in *R. ornithinolytica* N9, specifically in the pBK30683-like plasmid pN9KPC [49] (Figure 2). The mobile genetic platform *bla*_KPC-3_-Tn*4401* appears to be widespread in CRE isolated from Portuguese hospitals [10,93,94], and has also been detected circulating in the environment [11,14], associated with both IncN and FIA/FII plasmids. The Tn*4401* transposon has also been proven to have an important role in the dissemination of *bla*_KPC_-type genes within a large range of plasmid structures and bacterial species [18,95,96]. Although it was not possible to determine the type of plasmid structure in which the *bla*_KPC-3_ gene was located in *C. freundii* N6 and *E. kobei* N10, the replicons (e.g., IncN, IncX5, and pKPC-CAV1321) identified by the in silico analysis of both these isolates have already been associated with *bla*_KPC_ in both clinical [91,96,97] and environmental isolates [35,98], including in Portugal [8,11,94]. 

CRE harboring pBK30683-like plasmids have already been reported in Portugal, namely in clinical *K. pneumoniae* isolates [94] and in environmental *K. pneumoniae* isolated from a highly polluted river [11]. However, to the best of our knowledge, this is the first time that such plasmids have been described in *Raoultella* isolates.

*Raoultella* species are closely related to *Klebsiella* spp. and can be found ubiquitously in the environment, although they have already been associated with several cases of human infections linked to production of carbapenemases [99,100,101,102]. In addition, *Raoultella* isolates harboring carbapenemases have also been described in environmental settings, although never in Portugal [103,104,105,106]. Gomi and colleagues described the occurrence of *bla*_GES-5_ situated in a class 1 integron in a *R. ornithinolytica* strain isolated from wastewater [104]. In *R. ornithinolytica* N9, although this gene was likely present in chromosomal DNA, it was associated with a class 3 integron, which may play a potential role in the dissemination of this gene. *R. ornithinolytica*, previously known as *K. ornithinolytica*, is often misidentified as *K. oxytoca* by conventional biochemical identification methods [101,107]. Due to this common misidentification, the significance of *Raoultella* spp. as a nosocomial pathogen may be underestimated, as well as their association with carbapenemase production [108].

The role of the *Enterobacter* genus in carbapenemase gene flow between environmental, animal, and human settings has also been recognized [86,109]. *Enterobacter* isolates harboring *bla*_KPC_ and *bla*_GES_ genes have already been described in both clinical [110,111,112,113] and environmental settings [90,104].

In addition, in Portugal the *bla*_GES-5_ gene has already been found in association with *IntI3* in clinical *K. pneumoniae* isolates [92], and also in two *Citrobacter* environmental isolates [11,19]. The occurrence of *bla*_KPC-3_ in Tn*4401*b transposons in both *C. freundii* F6 and *E. kobei* N10 highlighted the importance of these MGEs in the dissemination and acquisition of carbapenemase genes and other ARGs among different *Enterobacterales* species [114]. This was also pointed out by the occurrence of plasmids harboring the *bla*_GES-5_ gene that exhibited a high similarity to the one detected in *E. kobei* N10, in different *Enterobacterales* hosts isolated from environmental [11,19] and clinical settings [20] (Figure 3). Furthermore, the association of the *bla*_GES-5_ gene described in *E. kobei* N10 with a class 3 integrase and a genomic island enhances the chances of horizontal gene-transfer events [115,116].

The results obtained here highlighted that, although urban ponds are usually smaller and more confined than other aquatic systems, they are associated with bacteria that have a large variety of ARGs and also MGEs, which contribute to the dissimilation of these genes. The important role of conjugative plasmids was made clear, as these structures were identified in all CRE isolates, including previously described plasmids that were associated with dispersion of carbapenemase genes in clinical settings. In addition to plasmids, a considerable variety of MGEs were also identified, including genomic islands, transposons, and integrons. The importance of integrons was evidenced here, as they were linked with MDR phenotypes (in strains *R. ornithinolytica* N9, *C. freundii* F6, and *Citrobacter* N5) and the dispersion of carbapenemase genes (in strains *R. ornithinolytica* N9 and *E. kobei* N10).

## 5. Conclusions

In conclusion, our study supported the evidence that urban landscape ponds can serve as hotspots for multidrug-resistant bacteria that are also resistant to last-resort antibiotics. It is also worrisome that the acquired carbapenemase genes described here were located in conjugative plasmids and at the same time were associated with other mobile genetic elements such as integrons and genomic islands.

The fact that CRE were detected in every sampling campaign and that the same strains were able to persist within these ponds over time represents a serious public health threat, and highlights the importance of adopting a One Health approach, in which the environment is viewed as a transmission vehicle of antimicrobial resistance. 

Further investigation should focus on elucidating the source(s) of contamination with CRE, particularly in Pond 1, from which most of the CRE isolates were obtained. Moreover, the transmission of resistance from these urban aquatic systems to humans and the underlying clinical implications are still largely underexplored and should also be attained.

## Figures and Tables

**Table 1:** Features of CRE obtained from the urban ponds (P) and estuarine canal (E).

								Resistance Profile ^b^															
Isolate	Sampling Site of Origin	Sampling Date	Phylogenetic Affiliation ^a^	Typing Profiles ^c^	Replicons	Integrase Gene	Carbapenemase Gene	PRL	TZP	FEP	CTX	CAZ	IPM	ETP	MEM	ATM	TE	TGC	AK	CIP	C	SXT	CN
F1	P1	Feb/19	*Citrobacter*	A-V	-	*IntI1*	*bla*_KPC_																
F2	P1		*Citrobacter*	A	-	*IntI1*	*bla*_KPC_																
F3	P1		*Citrobacter*	A-VII	IncN	*IntI1*	*bla*_KPC_																
F4	P1		*Citrobacter*	A-VI	IncL/M	*IntI1*	*bla*_KPC_																
F5	P1		*Citrobacter*	B-IX	-	*IntI1*	*bla*_KPC_																
F6	P1		*Citrobacter*	A-VIII	IncN; IncM1; IncFIA(HI1); IncFII(K); pKPC-CAV1321	*IntI1*	*bla*_KPC_																
S1	P1	Sept/19	*Raoultella*	D-I	-	*IntI1*	*bla*_KPC_																
S3	P1		*Raoultella*	D	-	*IntI1*	*bla*_KPC_																
S4	P1		*Raoultella*	D	-	*IntI1*	*bla*_KPC_																
S5	P1		*Raoultella*	D	-	*IntI1*	*bla*_KPC_																
O1	P1	Oct/19	*Raoultella*	D-I	-	*IntI1*	*bla*_KPC_																
O5	P1		*Raoultella*	D-II	-	*IntI1*	*bla*_KPC_																
N1	P1	Nov/19	*Raoultella*	E-III	IncFIA-FII	*IntI1*	*bla*_KPC_; *bla*_GES_																
N5	P1		*Citrobacter*	C-X	IncN	*IntI1*	*bla*_KPC_; *bla*_VIM_																
N6	P1		*Citrobacter*	A-V	IncN	*IntI1*	*bla*_KPC_																
N8	P1		*Raoultella*	E-III	IncL/M	*IntI1; IntI3*	*bla*_KPC_; *bla*_GES_																
N9	P1		*Raoultella*	E-III	IncFIA-FII; CoI(MGD2); CoI(pHAD28)	*IntI1; IntI3*	*bla*_KPC_; *bla*_GES_																
N10	P1		*Enterobacter*	G	CoI440I; IncFIB-FII; IncM1; IncN; IncX5; pKPC-CAV1321	*IntI1; IntI3*	*bla*_KPC_; *bla*_GES_																
N11	P3		*Enterobacter*	H	-	-	-																
N12	E1		*Raoultella*	E-IV	IncFIA-FII	*IntI1; IntI3*	*bla*_KPC_; *bla*_GES_																
N13	E1		*Raoultella*	E-III	-	*IntI1; IntI3*	*bla*_KPC_; *bla*_GES_																
N14	P1		*Klebsiella*	F	-	*IntI3*	*bla*_GES_																
N15	P3		*Enterobacter*	H	-	-	-																

^a^ Determined by BLAST using 16S rRNA gene. ^b^ PRL, piperacillin; TZP, piperacillin–tazobactam; FEP, cefepime; CTX, cefotaxime; CAZ, ceftazidime; IPM, imipenem; ETP, ertapenem; MEM, meropenem; ATM, aztreonam; TE, tetracycline; TGC, tigecycline; AK, amikacin; CIP, ciprofloxacin; C, chloramphenicol; SXT, trimethoprim–sulfamethoxazole; CN, gentamicin (dark grey, resistant; white, susceptible). ^c^ BOX profiles are designated with letters and PFGE profiles with Roman numerals; A hyphen is displayed whenever no replicon, carbapenemase gene, and integrase gene were detected.

**Table 2:** MICs of carbapenems and cephalosporins for *Citrobacter* isolates F1 and N6, transconjugants *E. coli* J53::KPC, and recipient strain *E. coli* J53.

	MIC, mg/L (Susceptibility)				
	ETP	MEM	IPM	CAZ	CTX
*E. Coli* J53	0.012 (S)	0.016 (S)	0.38 (S)	0.047 (S)	0.016 (S)
*Citrobacter* F1	>32 (R)	16 (R)	>32 (R)	>256 (R)	12 (R)
*E. coli* J53 (F1.1t)	0.125 (S)	0.5 (S)	2 (S)	12 (R)	3 (R)
*Citrobacter* N6	2 (R)	8 (IR)	>32 (R)	64 (R)	6 (R)
*E. coli* J53 (N6.1t)	0.75 (R)	0.5 (S)	8 (R)	32 (R)	4 (R)

ETP, ertapenem; MEM, meropenem; IPM, imipenem; CAZ, ceftazidime; CTX, cefotaxime (R, resistant; IR, intermediate; S, susceptible).

**Table 3:** Putative ARGs and plasmids predicted by CARD and PlasmidFinder, respectively, in CRE isolates.

	Isolates		
Drug Class/Predicted Plasmids	*C. freundii* F6	*R. ornithinolytica* N9	*E. kobei* N10
Beta-lactam resistance	*bla*_KPC-3_	*bla*_KPC-3_	*bla*_KPC-3_
	*bla*_OXA-1_	*bla*_GES-5_	*bla*_GES-5_
	*bla_CMY-152_*	*bla_ORN-1_*	*bla*_ACT-9_
		*bla*_MOX-3_	
		*bla*_OXA-9_	
		*bla_OXA-10_*	
		*bla_TEM-1_*	
Chloramphenicol resistance	*catB3*		
Aminoglycoside resistance	*aac(6′)-Ib-cr*	*aph(6)-Id* (s*trB*)	*aac(6′)-Ib9*
		*aacA4*	
		*aadA1*	
		*aph(3′’)-Ib* (s*trA*)	
Macrolides resistance	*mphA*	*mphE*	*mphA*
Quinolones resistance	*qnrS1*		
Sulfonamides resistance	*sul1*	*sul2*	*sul1*
Diaminopyrimidine resistance	*dfrA14*	*dfrA14*	
Tetracycline resistance	*tetA-like*		
Fosfomycin resistance		*fosA*	*fosA*
Resistance to other antibiotics		*msr(E)*	
Plasmid Content	IncFIA(HI1)	Col(MGD2)	CoI440I
	IncFII(K)	Col(pHAD28)	IncFIB(pECLA)
	IncM1	CoI440I	IncM1
	IncN	IncFIA/FII (pBK30683)	IncN
	pKPC-CAV1321		IncX5
			pKPC-CAV1321

## Data Availability

All relevant data are within the manuscript and its Supporting Material files. Additionally, the whole-genome sequences of *Citrobacter freundii* F6, *Raoultella ornithinolytica* N9, and *Enterobacter kobei* N10 can be found in the DDBJ/ENA/GenBank database under the accession numbers JAJBJE000000000, JAJDEY000000000, and JAKKZG00000000, respectively.

## Supplementary Materials

The following supporting information can be downloaded at: https://www.mdpi.com/article/10.3390/ijerph19105848/s1, Table S1: PCR primers used in molecular typing, 16S-rRNA-based affiliation, and screening of ARGs and integrons [76,117,118,119,120]. Table S2: Percentage of imipenem-resistant CFUs in the sampled ponds and estuarine canal in February and March 2019, and from September 2019 to February 2020. No imipenem-resistant colonies were obtained from Pond P4 and P5. Hyphens are exhibited whenever no imipenem-resistant CFUs were detected. Table S3: General features of *C. freundii* F6, *R. ornithinolytica* N9, and *E. kobei* N10 draft genomes, and WGS-based parameters used for phylogenetic affiliation. Figure S1: Average counting of typical coliforms in CFU/mL (log_10_) collected in February and March 2019, and from September 2019 to February 2020, from five ponds and an estuarine canal. In both February and March 2019, samples were only collected from ponds P1 and P2, while in September 2019, no samples were collected from E1. Figure S2: Dendrogram with the PFGE restriction patterns of genomic DNA from the eight Raoultella (A) and the seven Citrobacter (B) selected strains digested with XbaI. The dendrogram was constructed using GelCompar II version 6.1 (Applied Maths, Belgium) with a Dice coefficient and UPGMA clustering (Opt. 2.00%; Tol 1.0%; H > 0.0%; S > 0.0%). Figure S3: Plasmid DNA fingerprint of the entire CRE collection comprising 23 isolates. The molecular weight marker 1.5 Kb DNA NZYDNA Ladder VI (NzyTech, Portugal) is represented by the letter M. Figure S4: Circular genome representation of *C. freundii* F6 (A), *R. ornithinolytica* N9 (B), and *E. kobei* N10 (C). BRIG performed the alignment using a local BLAST+ with the standard parameters (50% lower-70% upper cut-off for identity and e-value of 10). The ring color gradients correspond to varying degrees of identity of BLAST matches. Circular genomic maps include information on GC skew, GC content, putative pathogenicity islands (PIs), putative resistance islands (RIs), prophage sequences (PS), and the location of ARGs and CRISPRs within the clockwise and counterclockwise CDS strands. Figure S5: Category of bacterial virulence factors found in *C. freundii* F6 (a), *R. ornithinolytica* N9 (b), and *E. kobei* N10 (c) according to the VFDB analysis.
