# Supplementary material for: KPC-3-, GES-5-, and VIM-1-Producing Enterobacterales Isolated from Urban Ponds
Source: Int J Environ Res Public Health. 2022 May 11;19(10):5848. doi: 10.3390/ijerph19105848 (PMC9141432; doi:10.3390/ijerph19105848)
Supplement: Supplementary file 1 [file ijerph-19-05848-s001.zip › ijerph-1685432-supplementary.pdf]

KPC-3, GES-5 and VIM-1-producing *Enterobacterales* isolated from urban ponds

Pedro Teixeira[1,2#], Nuno Pinto[2#], Isabel Henriques[3*], Marta Tacão[1,2]

[1]CESAM (Centre for Marine and Environmental Studies), University of Aveiro, Aveiro, Portugal

[2]Biology Department, University of Aveiro, Aveiro, Portugal

[3]University of Coimbra, Centre for Functional Ecology, Department of Life Sciences, Coimbra, Portugal

*Corresponding author: isabel.henriques@uc.pt

[#]Authors contributed equally

SUPPLEMENTARY MATERIAL

Tables S1-S3
Figures S1-S5

Table S1. PCR primers used in molecular typing, 16S rRNA based affiliation and screening of ARGs and Integrons

Gene	Sequences	Fragment size(bp)
*bla*KPC[76]	FW 5' - CATTCAAGGGCTTTCTTGCTGC<br>REV 5' - ACGACGGCATAGTCATTTGC	538
*bla*NDM[76]	FW 5' - GGTTTGGCGATCTGGTTTTC<br>REV 5' - CGGAATGGCTCATCACGATC	621
*bla*VIM[76]	FW 5' - GATGGTGTTTGGTCGCATATCG<br>REV 5' - GCCACGTTCCCCGCAGACG	475
*bla*GES[76]	FW 5' - AGTCGGCTAGACCGGAAAG<br>REV 5' - TTTGTCCGTGCTCAGGAT	399
*bla*OXA-48[76]	FW 5' - TTGGTGGCATCGATTATCGG<br>REV 5' - GAGCACTTCTTTTGTGATGGC	744
*bla*CTX-M[76]	FW 5' - GTGCAGTACCAGTAAAGTTATGG<br>REV 5' - CGCAATATCATTGGTGGTGCC	538
mcr-1[117]	FW 5' - CGGTCAGTCCGTTTGTTC<br>REV 5' - CTTGGTCGGTCTGTAGGG	350
16S rRNA[118]	FW 5' - AGAGTTTGATCCTGGCTCAG<br>REV 5' - GGTTACCTTGTTACGACTTT	1467
IntI1[119]	FW 5' - ACATGCGTGTAAATCGTC<br>REV 5' - CTGGATTTCGATGACGGCACG	280
IntI2[119]	FW 5' - ACGGCTACCCTCTGTTAT<br>REV 5' - TTATTGCTGGGATTAGGC	233
IntI3[119]	FW 5' - AGTGGGTGGCGAATGAGTG<br>REV 5' - TGTTCTTGTATCGGCAGGTG	600
BOX element[120]	BOXA1R: CTACGGCAAGGCGACGCTGAC	variable

Table S2. Percentage of imipenem resistant CFUs in the sampled ponds and estuarine canal in February and March 2019, and from September 2019 to February 2020. No imipenem-resistant colonies were obtained from Pond P4 and P5. Hyphens are exhibited whenever no imipenem resistant CFUs were detected.

Sampling sites	feb/19	mar/19	sept/19	oct/19	nov/19	dec/19	jan/20	feb/20
P1	0.0244	0.0018	0.1609	0.0058	0.0535	0.0045	0.0019	0.0018
P2	0.0003	0.0010	-	-	-	-	-	-
P3	-	-	-	-	0.0005	-	-	-
E1	-	-	-	0.0045	0.0624	0.0032	0.0047	0.0025

Table S3. General features of *C. freundii* F6, *R. ornithinolytica* N9 and *E. kobei* N10 draft genomes and WGS-based parameters used for phylogenetic affiliation.

	***C. freundii* F6**	***R. ornithinolytica* N9**	***E. kobei* N10**
Size (bp)	5,117,230	6,252,962	5,397,864
GC content (%)	51.7	55.2	54.4
Nº Contigs	230	420	431
Coverage	180x	170x	115x
N50	88,228	109,201	65,421
Nº of CDS	4997	6014	5128
Nº of tRNAs	80	84	80
Nº of rRNAs	2	2	1
Sequence Type	ST270	-	ST1378
ANIb (%)*	98.32	99.16	99.25
ANIm (%)*	98.98	99.53	99.85
dDDH(%)*	90.50	94.90	94.30
Difference in %G+C*	0.01	0.47	0.01

*For species identification, *C. freundii* ATCC 8090 (JMTA01000050), *R. ornithinolytica* NBRC 105727 (BCYR0100000000) and *E. kobei* DSM13645 (CP017181) were used as reference genomes.

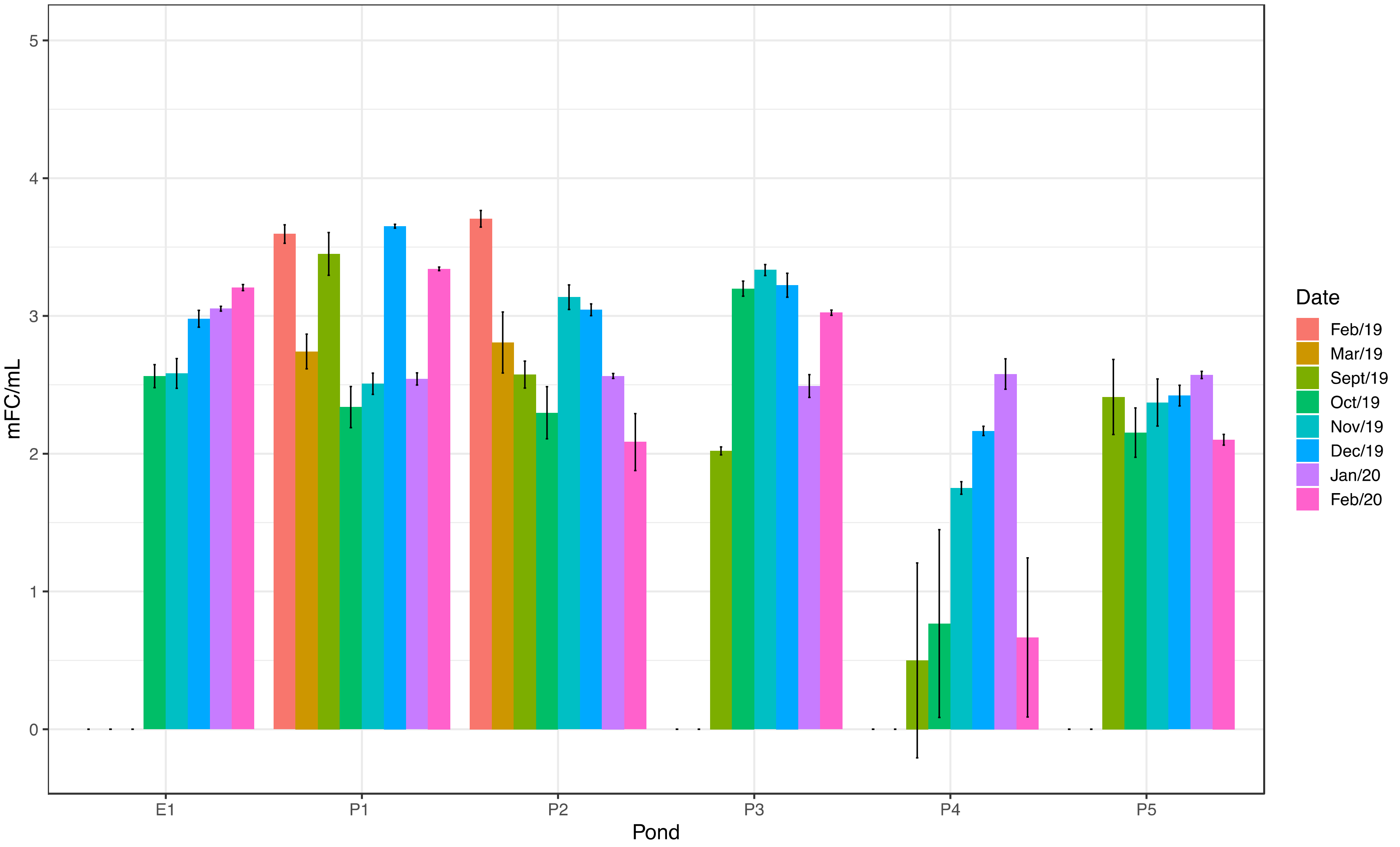

Figure S1. Average counting of typical coliforms in CFU/mL (log_{10}) collected in February and March 2019, and from September 2019 to February 2020, from five ponds and an estuarine canal. In both February and March 2019, samples were only collected from ponds P1 and P2, while in September 2019, no samples were collected from E1.

A

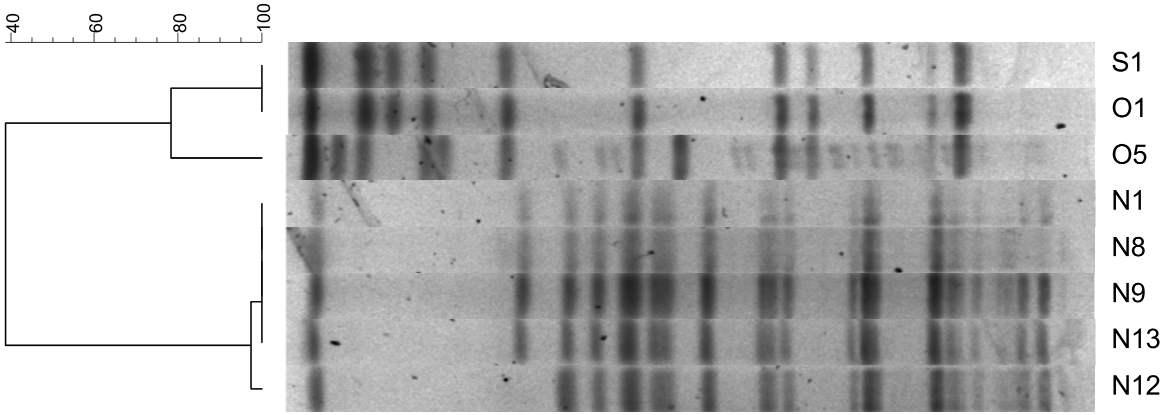

B

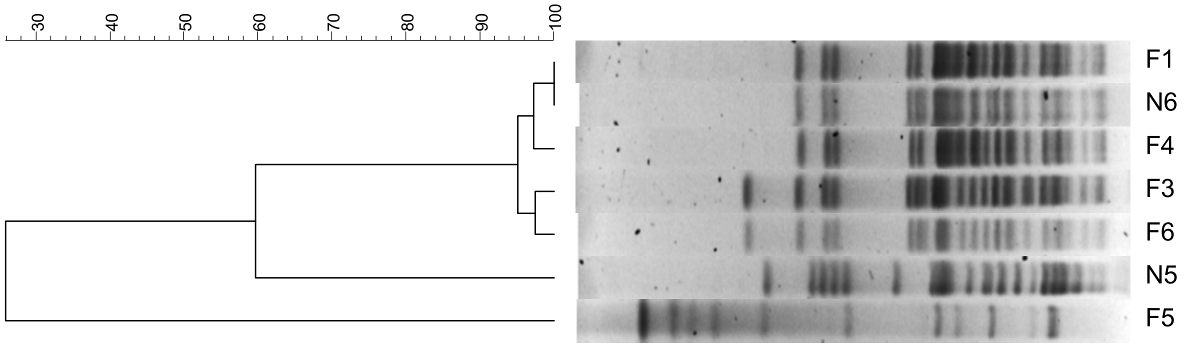

Figure S2. Dendrogram with the PFGE restriction patterns of genomic DNA from the 8 *Raoultella* (A) and the 7 *Citrobacter* (B) selected strains digested with XbaI. The dendrogram was constructed using GelCompar II version 6.1 (Applied Maths, Belgium) with Dice coefficient and UPGMA clustering (Opt. 2.00%; Tol 1.0%; H > 0.0%; S > 0.0%).

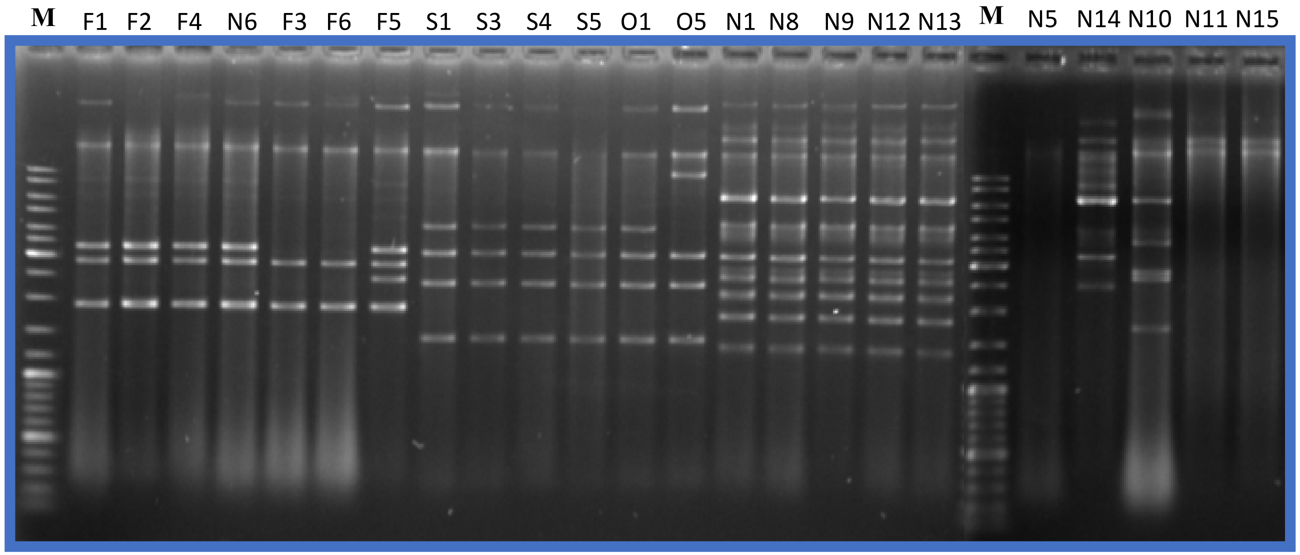

Figure S3. Plasmid DNA fingerprint of the entire CRE collection comprising 23 isolates. The molecular weight marker 1.5 Kb DNA NZYDNA Ladder VI (NzyTech, Portugal) is represented by the letter M.

A

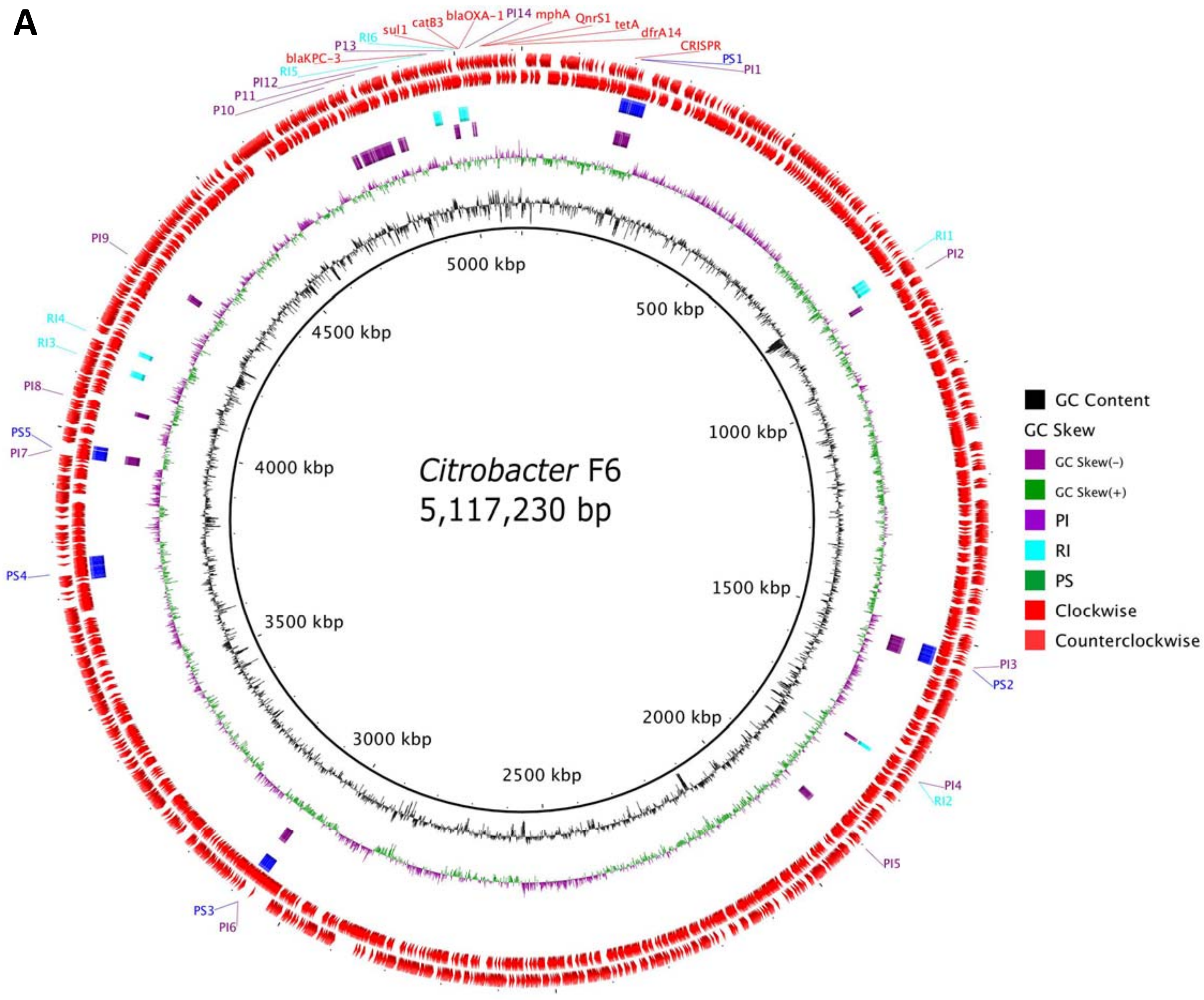

Citrobacter F6
5,117,230 bp
5000 kbp
500 kbp
1000 kbp
1500 kbp
2000 kbp
2500 kbp
3000 kbp
3500 kbp
4000 kbp
4500 kbp
sul1
catB3
blaOXA-1
PI14
mphA
QnrS1
tetA
dfrA14
CRISPR
PS1
PI1
RI6
P13
blaKPC-3
RI5
PI12
PI11
PI10
RI1
PI2
PI9
RI4
RI3
PI8
PS5
PI7
PS4
PI3
PS2
PI4
RI2
PI5
PS3
PI6
GC Content
GC Skew
GC Skew(-)
GC Skew(+)
PI
RI
PS
Clockwise
Counterclockwise

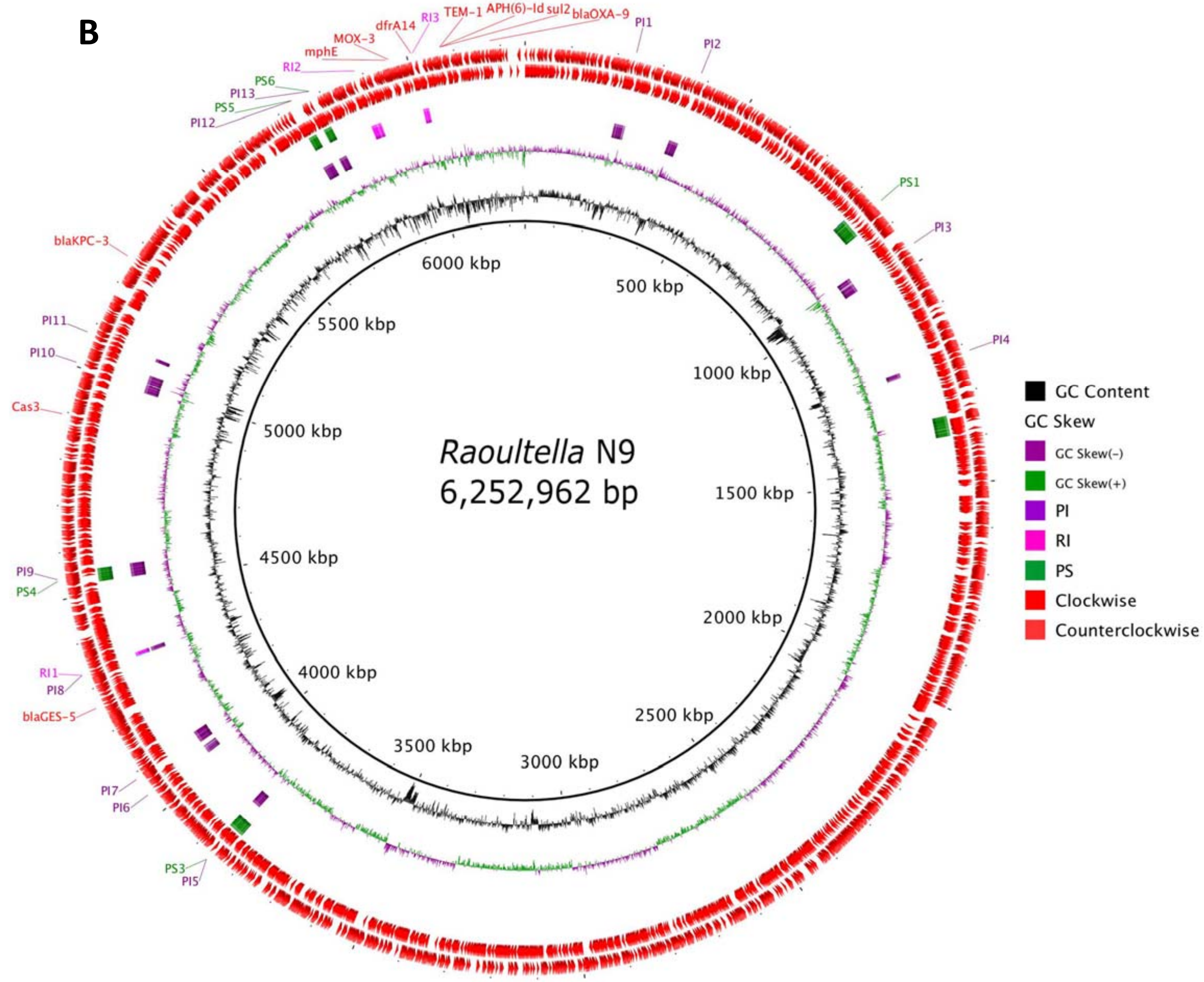
B
Raoultella N9
6,252,962 bp
500 kbp
1000 kbp
1500 kbp
2000 kbp
2500 kbp
3000 kbp
3500 kbp
4000 kbp
4500 kbp
5000 kbp
5500 kbp
6000 kbp
mphE
MOX-3
dfrA14
RI3
TEM-1
APH(6)-Id
sul2
blaOXA-9
PI1
PI2
RI2
PS6
PI13
PS5
PI12
PS1
PI3
blaKPC-3
PI4
PI11
PI10
Cas3
PI9
PS4
RI1
PI8
blaGES-5
PI7
PI6
PS3
PI5
GC Content
GC Skew
GC Skew(-)
GC Skew(+)
PI
RI
PS
Clockwise
Counterclockwise

C

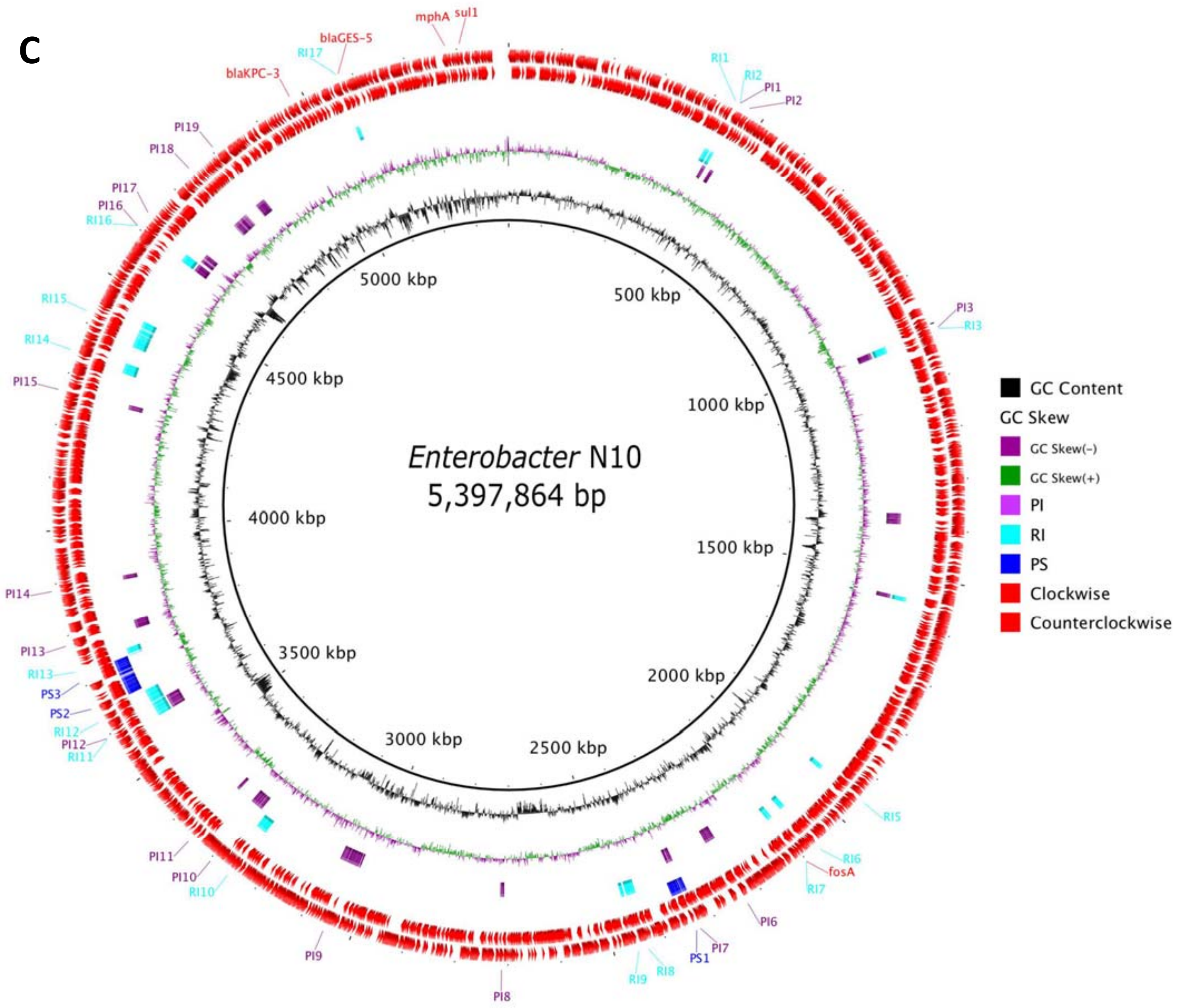
mphA
sul1
blaGES-5
RI17
blaKPC-3
RI1
RI2
PI1
PI2
PI19
PI18
PI17
PI16
RI16
RI15
RI14
PI15
PI3
RI3
5000 kbp
500 kbp
4500 kbp
1000 kbp
Enterobacter N10
5,397,864 bp
4000 kbp
1500 kbp
3500 kbp
2000 kbp
3000 kbp
2500 kbp
PI14
PI13
RI13
PS3
PS2
RI12
PI12
RI11
RI5
RI6
fosA
RI7
PI11
PI10
RI10
PI6
PI9
PI7
PS1
RI8
RI9
PI8
GC Content
GC Skew
GC Skew(-)
GC Skew(+)
PI
RI
PS
Clockwise
Counterclockwise

Figure S4. Circular genome representation of *C. freundii* F6 (A), *R. ornithinolytica* N9 (B) and *E. kobei* N10 (C). BRIG performed the alignment using a local BLAST+ with the standard parameters (50% lower-70% upper cut-off for identity and e-value of 10). The ring colour gradients correspond to varying degrees of identity of BLAST matches. Circular genomic maps include information on GC Skew, GC content, putative pathogenicity islands (PIs), putative resistance islands (RIs), prophage sequences (PS) and the location of ARGs and CRISPRs within the clockwise and counterclockwise CDS strands.

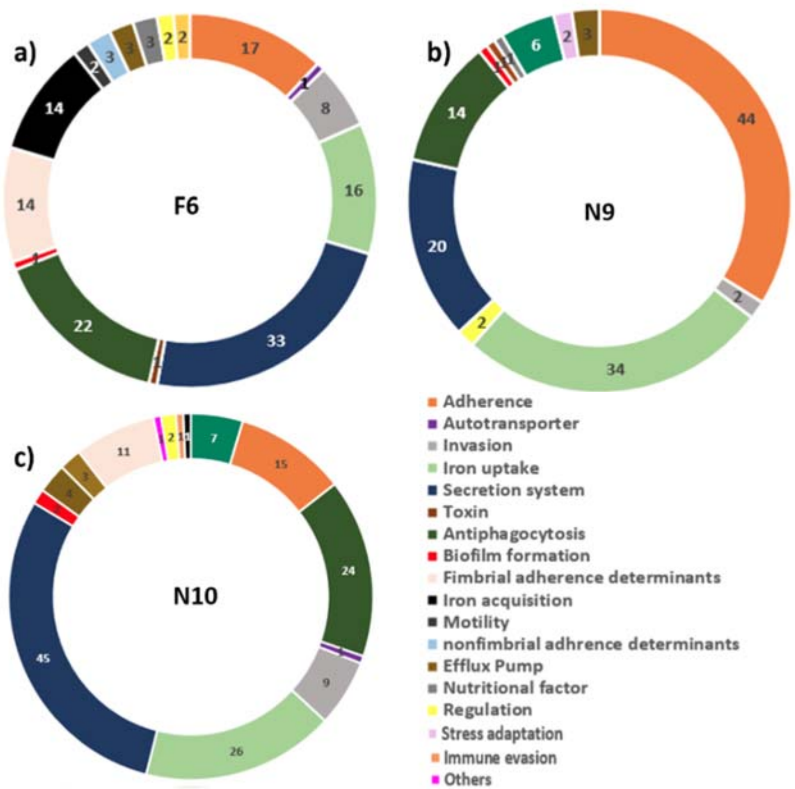

Figure S5. Category of bacterial virulence factors found in *C. freundii* F6 **a)**, *R. ornithinolytica* N9 **b)** and *E. kobei* N10 **c)** according to the VFDB analysis.
